# Development, Reliability, and Validity of the“Knowledge-Attitude-Practice” Questionnaire of Foreigners on Traditional Chinese Medicine Treatment

**DOI:** 10.1155/2020/8527320

**Published:** 2020-10-21

**Authors:** Lusha Li, Junlu Zhang, Qiaohua Qiao, Lihong Wu, Liying Chen

**Affiliations:** Department of General Practice, Sir Run Run Shaw Hospital, School of Medicine, Zhejiang University, Hangzhou 310016, China

## Abstract

**Objective:**

To develop a “knowledge-attitude-practice” questionnaire as an evaluating tool of foreigners' cognition on TCM treatment, so as to promote the internationalization of TCM.

**Methods:**

The questionnaire was based on the “knowledge-attitude-practice” model and adjusted by expert consultation using the Delphi method. After conducting a survey among foreigners, Cronbach's *α* and exploratory factor analysis were used to test the internal consistency reliability and structural validity of the questionnaire, respectively.

**Results:**

A total of 10 experts participated in two rounds of expert consultation. The recovery rates of two rounds of expert consultation form were 100.0%. The coefficient authority in two rounds of expert consultation was 0.87 and 0.88, respectively. The concentration of expert opinions in the knowledge, attitude, and practice dimensions was 3.80 to 4.70 points, 3.70 to 4.50 points, and 3.60 to 4.40 points, respectively, in the first round and 4.30 to 4.80 points, 4.10 to 4.60 points, and 4.00 to 4.50 points, respectively, in the second round. The coefficient of variation in the knowledge, attitude, and practice dimensions was 0.10–0.32, 0.16–0.29, and 0.19–0.35, respectively, in the first round and 0.09–0.19, 0.15–0.25, and 0.16–0.31, respectively, in the second round. The *W* value and significance test *x*^2^ in the first round were 0.657 and 218.620 while those in the second round were 0.671 and 181.181(*P* < 0.001). 8 items were deleted and 1 item was added, and other reserved items were modified according to the statistical analysis results of evaluation items and expert suggestions after the first round and there were no changes after the second round. The revised questionnaire includes three dimensions of knowledge, attitude, and practice, with a total of 30 items. After translating the questionnaire into English, it was conducted in 176 foreigners. Cronbach's *α* coefficient of the total questionnaire, knowledge dimension, attitude dimension, and practice dimension was 0.908, 0.781, 0.823, and 0.918, respectively. Exploratory factor analysis extracted 3 factors with a cumulative contribution of 54.090%. After testing reliability and validity, 1 item was deleted, leaving 29 items.

**Conclusions:**

After two rounds of expert consultation based on the Delphi method, the results of expert authority, expert coordination, and expert opinions' concentration were promising, and the expert consultation results were reliable. The “knowledge-attitude-practice” questionnaire of foreigners on TCM treatment in English had good reliability and validity and can evaluate foreigners' cognition on TCM treatment.

## 1. Introduction

Traditional Chinese medicine (TCM) had a long history in China and was a precious traditional culture of Chinese people. In the middle of the 19^th^ century, the British East India Company named Chinese medicine “TCM” in order to distinguish from Western medicine. Until 1936, the “regulations on TCM” made by the Kuomintang government officially established the name of “TCM” [1]. In 1996, China introduced the concept of “internationalization of TCM” for the first time [2], which was aimed at making TCM accessible to the world [3]. Up to now, TCM had spread to 183 countries and regions in the world. According to the World Health Organization (WHO), 103 member states had approved the use of acupuncture [1]. As reported by Overview of International Traditional Chinese Medicine Development and Legislation [4], there were more than 1,500 TCM clinics in the Netherlands, more than 3,000 TCM clinics in the UK, more than 3,000 acupuncture clinics in France, more than 3,000 TCM clinics in Canada, and more than 2,500 TCM clinics in Australia.

However, after more than 2 decades of efforts to the internationalization of TCM, the progress appeared to be stagnated [2]. Although the scale of TCM development was growing, the depth of development was not enough. Many people blindly denied the scientific nature of TCM and questioned the standardization of TCM diagnosis and treatment system, which made TCM difficult to be trusted by more people [5]. Although acupuncture and moxibustion were increasingly valued in many countries because they were relatively easy to learn and had been proven efficient in treating painful diseases, Chinese medicine was restricted or banned in some countries because of the difficulty in learning prescriptions as well as the exceeding content of toxicity, pesticide, and heavy metal in some Chinese medicine. As an important part of TCM treatment, massage was in a more marginal state compared with these treatments in many countries [4].

There were limited studies on the challenges facing the internationalization of TCM [2]. Some studies had pointed out the reasons hindering the further internationalization of TCM, such as the difficulty to understand the true connotation of TCM [3], the huge barriers between countries' trade [5], the lacking of the strict industrial standard [5], the unscientific export structure [5], the limited input in research [3], and the absence of medical insurance [4]. The “knowledge-attitude-practice” (KAP) questionnaire was generally used to understand the knowledge, attitude, and practice of the target population in health care as well as the demand and acceptance degree of relevant content [6]. Up to now, there was no study of foreigners' knowledge, attitude, and practice on TCM treatment. Our research group developed a KAP questionnaire as an evaluating tool of foreigners' cognition on TCM treatment, which can further promote the internationalization of TCM.

## 2. Method

**Figure 1:**
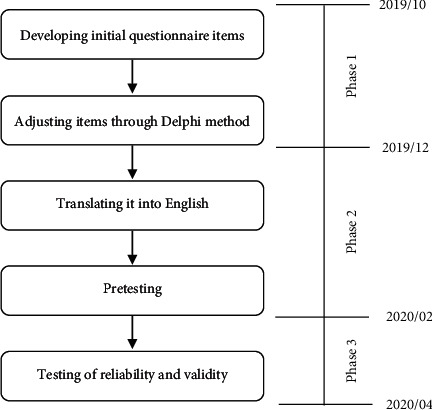
Stages of developing the KAP questionnaire of foreigners on TCM treatment in English.

This study included three phases. Phase 1 was from October to November 2019. The questionnaire was based on the “knowledge-attitude-practice” model and adjusted by expert consultation using the Delphi method. Phase 2 was conducted in December 2019. The questionnaire was translated into English and further revision to the questionnaire items was made following a pretest in 20 participants. Phase 3 was a formal survey to test the reliability and validity of the questionnaire from February to March 2020 (Figure 1).

### 2.1. Developing the KAP Questionnaire Items

Using the “knowledge-attitude-practice” model as the theoretical framework, the initial KAP questionnaire was developed on the base of a large number of domestic and foreign literatures on TCM, focus group discussions, as well as interviews with TCM experts.

### 2.2. Adjusting the KAP Questionnaire Items through Delphi Method

Through exchanging information and revising feedback many times, the Delphi method finally turned the expert opinions into the group consensus [7] and was used to develop evaluation index and scale [8]. After developing the initial KAP questionnaire, the Delphi method was adopted to adjust and confirm the questionnaire items.

#### 2.2.1. Expert Consultation Group

Expert inclusion criteria were as follows: (1) engaged in TCM-related work for more than 5 years with rich theoretical knowledge, diagnosis, and treatment background as well as practical experience; (2) intermediate professional title and above (doctoral graduate students, after assessment, can be directly identified as intermediate; master degree holders or double degree holders, after engaging in professional and technical work for more than 2 or 3 years, can be identified as intermediate; university graduate, after engaging in professional and technical work for more than 5 years, can be identified as intermediate); and (3) willing to participate in this expert consultation.

#### 2.2.2. Expert Consultation Process

Firstly, expert consultation form was sent by e-mail to 10 experts from Sir Run Run Shaw Hospital who met the inclusion criteria . Secondly, all the outcomes were summarized and analyzed, the item of initial KAP questionnaire inclusion criteria: (1) importance value of the item was ≥4.0 points; (2) coefficient of variation <0.25; (3) item which had no suggestion or question from experts; and (4) item which had no objection from the research group (item which met one of these four criteria can be included in the questionnaire) [6]. Based on the inclusion criterion, the items of the initial KAP questionnaire were adjusted according to experts' suggestions. Thirdly, all adjustments were summarized and illustrated, and then the new expert consultation form including the revised KAP questionnaire was sent to experts. Until the opinions of all experts tend to be the same, the KAP questionnaire was confirmed. In most studies, two rounds of expert consultation were used.

#### 2.2.3. Expert Consultation Form

The expert consultation form included 4 parts: introduction of the research background, instruction for filling in the form, general information of expert, and the initial KAP questionnaire.

The 5-point Likert rating method was used to determine the importance degree of items, which was set as “very important, important, neutral, unimportant, and very unimportant” and was rated as 5.0 points, 4.0 points, 3.0 points, 2.0 points, and 1.0 point, respectively. Expert familiarity degree was set as “very familiar, familiar, neutral, unfamiliar, and very unfamiliar” and was rated as 1.0 point, 0.8 points, 0.5 points, 0.2 points, and 0.0 points, respectively. Expert was supposed to judge items based on the theoretical analysis, practical experience, domestic and foreign references, and subjective intuition, each of which can be divided into “large, medium, and small” according to the influence degree of judgment. The sum of these four parts' scores formed the judgment coefficient (Table 1). And suggestions for the initial KAP questionnaire can be written in the revision section of the form.

### 2.3. Research on Reliability and Validity of the KAP Questionnaire

#### 2.3.1. Participants and Study Setting

In the attitude dimension, 4 items (A1, A2, A3, and A4) were not required for foreigners who had not received TCM treatment. In the practice dimension, 2 items (P8 and P9) were not required for male foreigners. These 6 items were not suitable for result statistics and analysis, leaving 24 items. According to the sample, size should be 5–10 times of the number of the questionnaire items, and 20% of the sample size should be expanded after considering factors such as invalidity [9], and the final calculated sample size was at least (24 × 5) × (1 + 20%) = 144.

The KAP questionnaire was used to conduct a survey of foreigners in Sir Run Run Shaw Hospital by the convenience sampling method. To make it more specified and reliable, we made some inclusion criteria for foreigners enrolled in our study, including comprehension of the content of the questionnaire and voluntary participation in this questionnaire. The exclusion criterion was participants who had serious illnesses, so that unable to complete the survey. All participants provided informed consent. The study was approved by the Ethics Committee of Sir Run Run Shaw Hospital, School of Medicine, Zhejiang University.

Some participants filled in paper questionnaires on site, while others filled in electronic questionnaires on mobile phones after scanning the QR code. Before conducting the survey, a trained researcher gave instructions to the participants. In the process of filling in the questionnaire, no other explanation was given. For participants who have chosen the electronic questionnaire, the same instructions were placed on the front page. Paper questionnaires were collected on site and checked by the researcher. If there were any incomplete answers, participants were asked to clarify. Electronic questionnaires were checked by the researcher from backstage and invalid questionnaires would be eliminated.

#### 2.3.2. Grading Method

A common grading method was used for each dimension in the KAP questionnaire; 3-point Likert rating method was used for knowledge and attitude dimensions, which was set as “agree, not sure, and disagree” and was rated as 3.0 points, 2.0 points, and 1.0 point, respectively, and 4-point Likert method was used for practice dimension, which was set as “often, sometimes, occasionally, and never” and was rated as 4.0 points, 3.0 points, 2.0 points, and 1.0 point, respectively.

#### 2.3.3. Pretest

In order to check whether the expression of items could be understood, the KAP questionnaire was piloted among 20 participants on site.

#### 2.3.4. Formal Survey

The survey was carried out from February to March 2020. Altogether, 176 questionnaires were collected anonymously.

### 2.4. Statistical Analysis

The data were entered into EpiData 3.1 Entry for data documentation. SPSS 23.0 and Excel 2003 were applied to further analyze the data. Data were presented as the mean ± standard deviation or *n* (%). Cronbach's *α* coefficient was applied to evaluate the reliability of the KAP questionnaire. Exploratory factor analysis was used to assess construct validity. The Kaiser–Meyer–Olkin (KMO) measure of sampling adequacy and Bartlett test of sphericity were used to assess the suitability of the data for factor analysis. *P* < 0.05 was considered statistically significant.

## 3. Results

### 3.1. Phase 1: Developing the KAP Questionnaire

#### 3.1.1. Focus Group Discussions

There were three rounds of focus group discussions and a total of 8 members of the research group were joined in. All participants felt free to talk openly and gave honest opinions, who not only expressed their own opinions but also responded to other members and questions posed by the leader.  Table 2 shows that 4 (50.0%) were females, 4 (50.0%) were 41–50 years old, 6 (75.0%) had a master's degree, and 6 (75.0%) majored in TCM.

#### 3.1.2. Initial KAP Questionnaire

The initial KAP questionnaire (Table 3) was determined and formed with three dimensions of knowledge, attitude, and practice and a total of 37 items.

#### 3.1.3. Expert Consultation Based on Delphi Method

In this study, we adopted two rounds of expert consultation, enrolling a total of 10 experts. The result showed that 9 (90.0%) were females, 7 (70.0%) were 31–40 years old, 7 (70.0%) had a master's degree, 8 (80.0%) majored in TCM, 5 (50.0%) were associate chief physicians, and 5 (50%) worked for 11 to 20 years. The detailed information is listed in Table 4. The recovery rates of expert consultation form were 100% in two rounds of expert consultation. And the coefficient authority in two rounds of expert consultation was 0.87 and 0.88, respectively. It was generally composed of expert judgment coefficient and expert familiarity degree, ranging from 0 to 1.00. The data were calculated based on the following formula: the coefficient authority of expert = (expert judgment coefficient + expert familiarity degree)/2.

To judge the degree of expert opinion coordination, Kendall's *W* was adopted. The value of Kendall's *W* was between 0 and 1, and the higher the value means, the opinion of the experts was more consistent and the coordination of the questionnaire was better [10]. In the first round, the *W* value was 0.657 (significance test *X*^2^ = 218.620,*P* < 0.001). In the second round, the *W* value was 0.671 (significance test *X*^2^ = 181.181,*P* < 0.001). After two rounds of expert consultation, the experts' opinion on each item tends to be consistent.

The degree of concentration of expert opinions was analyzed by the importance value and coefficient of variation. The greater the importance assignment, the smaller the coefficient of variation value indicated that the degree of expert opinion concentration was better [10]. The importance value in the knowledge, attitude, and practice dimensions was 3.80 to 4.70 points, 3.70 to 4.50 points, and 3.60 to 4.40 points, respectively, in the first round and 4.30 to 4.80 points, 4.10 to 4.60 points, and 4.00 to 4.50 points, respectively, in the second round. The coefficient of variation in the knowledge, attitude, and practice dimensions was 0.10–0.32, 0.16–0.29, and 0.19–0.35, respectively, in the first round and 0.09–0.19, 0.15–0.25, and 0.16–0.31, respectively, in the second round. And the detailed information is listed in Table 5. Compared to the first round, the importance value of items in the second round was larger while the coefficient of variation value was smaller.

In the first round of expert consultation, the research group deleted 8 items, modified 5 items, and added 1 item based on the inclusion criterion and experts' suggestions. And there were no changes in items after the second round. Thus, the revised KAP questionnaire of foreigners on TCM treatment was confirmed, which included 30 items.

### 3.2. Phase 2: Translation and Pretest

The revised KAP questionnaire was translated into English by 3 experts who had not only a background in TCM but also a good level of translating Chinese into English. In order to avoid bias, one of these experts was not part of the research group. They did discussions many times and generated the unified English version of the KAP questionnaire. Then this KAP questionnaire was piloted among 20 foreigners, and there was no adjustment after pretesting. The formal KAP questionnaire (Table 6) was formed with three dimensions of knowledge, attitude, and practice and a total of 30 items.

### 3.3. Phase 3: Reliability and Validity Analysis

#### 3.3.1. General Information of Participants

After removing 4 invalid questionnaires, there were 176 useful questionnaires, with a response rate of 97.78%. Of the participants, 90 (51.1%) were males, 103 (58.5%) were 20–39 years old, and the other general information is summarized in Table 7.

#### 3.3.2. Reliability Analysis

Cronbach's *α* coefficient was 0.908 overall, 0.781 for the knowledge dimension, 0.823 for the attitude dimension, and 0.918 for the practice dimension, indicating good internal consistency reliability.

#### 3.3.3. Validity Analysis

Construct validity was evaluated using exploratory factor analysis. In the Kaiser–Meyer–Olkin (KMO) test, the *r* value of 0.872 indicated that the data definitely lend itself to factor analysis. A Bartlett test of sphericity (*X*^2^ = 2234.69, df = 276, *P*=0.000) indicated that the analysis model was appropriate. For these reasons, it was acceptable to adopt factor analysis to test the construct reliability of this questionnaire [11, 12].

**Figure 2:**
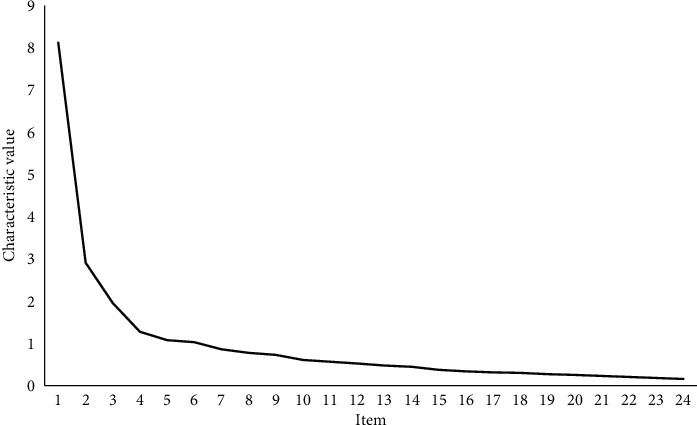
Gravel figure of questionnaire items.

Under the condition of unrestricted extraction factor values, a total of 6 factors with the characteristic value larger than 1 were extracted, and the accumulated contribution rate was 68.189%. The common factors were extracted by the principal component analysis method and rotated by the maximum difference method. Gravel figure (Figure 2) showed that the slope line gradually flattens after the fourth factor, and 3 dimensions were preset on the questionnaire. The cumulative variance contribution rate of these 3 factors was 54.090%. As shown in Table 8, after using exploratory factor analysis, item P11 should belong to the attitude dimension. Considering that item P11 was definitely the item related to behavior, in a further survey, it should be deleted because of the contradiction.

## 4. Discussion

The present study had developed a KAP questionnaire of foreigners on TCM treatment in English with good internal consistency reliability and construct validity. It was hoped that the application of this questionnaire would further our understanding of how TCM treatment affected foreigners and facilitated future promotion in the internationalization of TCM.

The brevity and understandability of the item likely facilitated the completion of the questionnaire by participants [12]. The number of items in a questionnaire had a significant influence on the quality of responses, and a time limit of 15–30 min was ideal [12]. The formal questionnaire contained a total of 29 items so that participants can be ensured to complete it about 20 minutes.

The questionnaire was scientific and authoritative to some extent since it was build based on the Delphi method. The Delphi method differed from traditional surveys in that panelists receive feedback as to how the panel was responding throughout the study. This encouraged them to consider their own responses alongside those of the entire panel and clarified or amended their responses based on this information [13]. Choosing appropriate experts was the key to the success of the Delphi method [14]. According to relevant studies, the coefficient authority of expert ≥0.80 indicated that experts had a good grasp of the choice of item [15]. As showed in the results, the coefficient authority in two rounds of expert consultation was 0.87 and 0.88, respectively, indicating that the invited experts had a high degree of authority in TCM and the consultation results were reliable. Thus, with the Delphi method and the high quality of experts, the questionnaire was rather scientific and dependable.

The reliability of the questionnaire was evaluated as internal consistency reliability. Cronbach's *α* represented the internal consistency of the questionnaire which showed whether the items in the same group were testing the same concept [16]. Many researchers maintained that Cronbach's *α* coefficient ≥0.7 indicated good internal consistency [11,12]. In this study, Cronbach's *α* coefficient of the questionnaire's total score was 0.908. Cronbach's *α* coefficient was 0.781 for the knowledge dimension, 0.823 for the attitude dimension, and 0.918 for the practice dimension. The above results showed that the questionnaire displayed good internal consistency, whether considering overall contents or individual dimensions. The overall content of the questionnaire can reflect an accurate portrayal of foreigners' cognition of TCM treatment while the contents of each dimension can accurately reflect the corresponding elements of TCM treatment.

Construct validity was used to evaluate whether or not the questionnaire corresponded to the theoretical design upon which the questionnaire was based. In this study, by analyzing the items within each common factor, we found that items P6, P2, P5, P3, P7, P4, P1, P12, and P10 reflected the practice dimension, items A3, A10, A2, A9, P11, A1, A4, and A11 reflected the attitude dimension, and items K4, K5, K6, K2, K7, K3, and K1 reflected the knowledge dimension. Results of the analysis showed that the structure of this scale was in accordance with the theoretical framework and original design except for item P11. Item P11 reflected the behavior of recommending TCM to others rather than attitude. Considering this contradiction, item P11 was deleted from the questionnaire, leaving 29 items.

Overall, the KAP questionnaire of foreigners on TCM treatment was simple and convenient and had relatively strong functionality and operability. Our study had several limitations. The experts selected in this study were clinical doctors working in hospitals. Although they had a good TCM background and most of them also taught in medical schools, their career distribution was relatively single. The participants selected in this study were only from a Grade A Class Three hospital in Hangzhou, and the representativeness of the sample was limited. The questionnaire should have been administered again in the same group of participants after at least 2 weeks to ensure test-retest reliability. However, due to the mobility of foreigners in hospitals, doing retest was less feasible. The reliability of the questionnaire was evaluated as internal consistency reliability. In future study, multicenter large sample surveys should be conducted in foreign communities, so that the representativeness of the sample can be increased. Meanwhile, the reliability and validity of the questionnaire should be further analyzed by expanding the sample size. After preliminarily applying the questionnaire in foreign communities, we can evaluate foreigners' cognition and application of TCM treatment, explore the relationship between foreigners' knowledge, attitude, and practice of TCM treatment, and find some influencing factors, so that to provide some suggestions for promoting the internationalization of TCM.

## 5. Conclusion

The “knowledge-attitude-practice” questionnaire of foreigners on traditional Chinese medicine treatment in English had been built using the Delphi method based on a panel of qualified experts. After two rounds of expert consultation, the results of expert authority, expert coordination, and expert opinions' concentration were promising, indicating the construction of the questionnaire was scientific. And the questionnaire had good reliability and validity, which can be used to evaluate foreigners' knowledge, attitude, and practice on TCM treatment.

## Figures and Tables

**Table 1:** Judgment basis of expert consultation based on the Delphi method.

Basis for judgment	Degree of influence on expert judgment		
	Large	Medium	Small
Theoretical analysis	0.3	0.2	0.1
Practical experience	0.5	0.4	0.3
Domestic and foreign references	0.1	0.1	0.1
Subjective intuition	0.1	0.1	0.1

**Table 2:** General information of participants (*n* = 8).

Item		*n*	%
Sex	Female	4	50.0
	Male	4	50.0

Age (years)	31–40	3	37.5
	41–50	4	50.0
	51–60	1	12.5

Education	Master's degree	6	75.0
	Doctor's degree	2	25.0

Major	Traditional Chinese medicine	6	75.0
	General practice medicine	2	25.0

**Table 3:** Initial “knowledge-attitude-practice” questionnaire of foreigners on TCM treatment.

*Knowledge*
K1. Traditional Chinese medicine is extensive and profound
K2. Traditional Chinese medicine is the Chinese traditional culture
K3. Traditional Chinese medicine is effective in treating chronic disease
K4. Traditional Chinese medicine is effective in treating acute disease
K5. Traditional Chinese medicine is effective in treating severe disease
K6. Chinese medicine is one of the treatments of traditional Chinese medicine
K7. Acupuncture and moxibustion is one of the treatments of traditional Chinese medicine
K8. Cupping is one of the treatments of traditional Chinese medicine
K9. Massage is one of the treatments of traditional Chinese medicine

*Attitude*
A1. You are willing to treat the disease by drinking Chinese medicines
A2. You are willing to treat the disease by receiving acupuncture and moxibustion
A3. You are willing to treat the disease by cupping
A4. You are willing to treat the disease by doing a massage
Note: if you have not received TCM treatment, please go to A9
A5. You are satisfied with the model of diagnosis and treatment of traditional Chinese medicine
A6. You are satisfied with the therapeutic effect of Chinese medicines
A7. You are satisfied with the therapeutic effect of acupuncture and moxibustion
A8. You are satisfied with the therapeutic effect of cupping
A9. You are satisfied with the therapeutic effect of massage
A10. You trust the therapeutic effect of traditional Chinese medicine
A11. Traditional Chinese medicine should be publicized and prompted
A12. Evidence-based research in traditional Chinese medicine should be strengthened

*Practice*
P1. When you suffer from chronic diseases, you will choose traditional Chinese medicine
P2. When you suffer from acute diseases, you will choose traditional Chinese medicine
P3. When you suffer from severe diseases, you will choose traditional Chinese medicine
P4. When you have headache and vertigo disease, you will choose traditional Chinese medicine
P5. When you suffer from chest tightness and shortness of breath, you will choose traditional Chinese medicine
P6. When you suffer from insomnia, you will choose traditional Chinese medicine
P7. When you suffer from diarrhea and constipation, you will choose traditional Chinese medicine
P8. When you suffer from sterility and infertility, you will choose traditional Chinese medicine
P9. When you suffer from hands ache or feet ache, you will choose traditional Chinese medicine
P10. When you have a bad appetite, you will choose traditional Chinese medicine
P11. When you are tired, you will choose traditional Chinese medicine
Note: if you are male, please go to P14
P12. When you suffer from menstruation disorder and dysmenorrhea, you will choose traditional Chinese medicine
P13. When you suffer from other gynecological diseases, you will choose traditional Chinese medicine
P14. You would like to attend the lecture about traditional Chinese medicine
P15. You would like to recommend traditional Chinese medicine to others
P16. You would like to use health tea or medicinal diet made by Chinese medicines

**Table 4:** General information of experts (*n* = 10).

Item		*n*	%
Sex	Female	9	90.0
	Male	1	10.0

Age (years)	31–40	7	70.0
	41–50	2	20.0
	51–60	1	10.0

Education	Bachelor's degree	1	10.0
	Master's degree	7	70.0
	Doctor's degree	2	20.0

Major	General practice medicine	2	20.0
	Traditional Chinese medicine	8	80.0

Title	Attending physician	4	40.0
	Associate chief physician	5	50.0
	Chief physician	1	10.0

Work time (years)	5–10	3	30.0
	11–20	5	50.0
	21–30	1	10.0
	31–40	1	10.0

**Table 5:** Result of the degree of expert opinions' concentration.

Item	Importance value (m ± sd)		Coefficient of variation (sd/m)	
	First round	Second round	First round	Second round
K1. Traditional Chinese medicine is extensive and profound	4.50 ± 0.53	4.70 ± 0.48	0.12	0.10
K2. Traditional Chinese medicine is the Chinese traditional culture	4.50 ± 0.53	4.60 ± 0.70	0.12	0.15
K3. Traditional Chinese medicine is effective in treating chronic disease	4.60 ± 0.70	4.80 ± 0.42	0.15	0.09
K4. Traditional Chinese medicine is effective in treating acute disease	3.90 ± 1.20	—	0.31	—
K5. Traditional Chinese medicine is effective in treating severe disease	3.80 ± 1.23	—	0.32	—
K6. Chinese medicine is one of the treatments of traditional Chinese medicine	4.70 ± 0.48	4.80 ± 0.42	0.10	0.09
K7. Acupuncture and moxibustion is one of the treatments of traditional Chinese medicine	4.50 ± 0.53	4.70 ± 0.48	0.12	0.10
K8. Cupping is one of the treatments of traditional Chinese medicine	4.10 ± 0.88	4.30 ± 0.82	0.21	0.19
K9. Massage is one of the treatments of traditional Chinese medicine	4.40 ± 0.70	4.60 ± 0.70	0.16	0.15
A1. You are willing to treat the disease by drinking Chinese medicines	4.50 ± 0.71	4.50 ± 0.71	0.16	0.16
A2. You are willing to treat the disease by receiving acupuncture and moxibustion	4.10 ± 0.88	4.40 ± 0.84	0.21	0.19
A3. You are willing to treat the disease by cupping	4.00 ± 0.94	4.60 ± 0.70	0.24	0.15
A4. You are willing to treat the disease by doing a massage	4.10 ± 0.88	4.20 ± 1.03	0.21	0.25
A5. You are satisfied with the model of diagnosis and treatment of traditional Chinese medicine	4.30 ± 0.82	4.10 ± 0.88	0.19	0.21
A6. You are satisfied with the therapeutic effect of Chinese medicines	4.00 ± 1.05	4.20 ± 0.92	0.26	0.22
A7. You are satisfied with the therapeutic effect of acupuncture and moxibustion	4.10 ± 0.88	4.20 ± 0.79	0.21	0.19
A8. You are satisfied with the therapeutic effect of cupping	3.70 ± 1.06	—	0.29	—
A9. You are satisfied with the therapeutic effect of massage	4.10 ± 0.88	4.40 ± 0.70	0.21	0.16
A10. You trust the therapeutic effect of traditional Chinese medicine	4.40 ± 0.84	4.40 ± 0.70	0.19	0.16
A11. Traditional Chinese medicine should be publicized and prompted	4.30 ± 1.06	4.30 ± 0.82	0.25	0.19
A12. Evidence-based research in traditional Chinese medicine should be strengthened	4.40 ± 0.70	4.40 ± 0.84	0.16	0.19
P1. When you suffer from chronic diseases, you will choose traditional Chinese medicine	4.20 ± 0.92	4.50 ± 0.71	0.22	0.16
P2. When you suffer from acute diseases, you will choose traditional Chinese medicine	3.60 ± 1.26	—	0.35	—
P3. When you suffer from severe diseases, you will choose traditional Chinese medicine	3.70 ± 1.16	—	0.31	—
P4. When you have headache and vertigo disease, you will choose traditional Chinese medicine	3.90 ± 1.10	—	0.28	—
P5. When you suffer from chest tightness and shortness of breath, you will choose traditional Chinese medicine	3.60 ± 1.07	—	0.30	—
P6. When you suffer from insomnia, you will choose traditional Chinese medicine	4.40 ± 0.84	4.50 ± 0.71	0.19	0.16
P7. When you suffer from diarrhea and constipation, you will choose traditional Chinese medicine	4.20 ± 1.03	4.30 ± 0.95	0.25	0.22
P8. When you suffer from sterility and infertility, you will choose traditional Chinese medicine	4.20 ± 0.79	4.40 ± 0.70	0.19	0.16
P9. When you suffer from hands ache or feet ache, you will choose traditional Chinese medicine	3.90 ± 1.10	—	0.28	—
P10. When you have a bad appetite, you will choose traditional Chinese medicine	4.30 ± 1.06	4.20 ± 1.03	0.25	0.25
P11. When you are tired, you will choose traditional Chinese medicine	4.30 ± 1.06	4.30 ± 1.06	0.25	0.25
P12. When you suffer from menstruation disorder and dysmenorrhea, you will choose traditional Chinese medicine	4.20 ± 1.03	4.50 ± 0.71	0.25	0.16
P13. When you suffer from other gynecological diseases, you will choose traditional Chinese medicine	4.00 ± 1.25	4.40 ± 0.97	0.31	0.22
P14. You would like to attend the lecture about traditional Chinese medicine	4.10 ± 1.10	4.50 ± 0.85	0.27	0.19
P15. You would like to recommend traditional Chinese medicine to others	4.10 ± 1.20	4.40 ± 0.84	0.29	0.19
P16. You would like to use health tea or medicinal diet made by Chinese medicines	4.10 ± 1.20	4.40 ± 0.84	0.29	0.19
P17. When you suffer from chills and fever, you will choose traditional Chinese medicine	—	4.00 ± 1.25	—	0.31

Note: —: did not have data.

**Table 6:** “Knowledge-attitude-practice” questionnaire of foreigners on TCM treatment.

*Knowledge*
K1. Traditional Chinese medicine is extensive and profound
K2. Traditional Chinese medicine is the Chinese traditional culture
K3. Traditional Chinese medicine is effective in treating chronic disease
K4. Traditional Chinese medicine treatments include Chinese medicine
K5. Traditional Chinese medicine treatments include acupuncture and moxibustion
K6. Traditional Chinese medicine treatments include cupping
K7. Traditional Chinese medicine treatments include massage

*Attitude*
A1. You are willing to treat the disease by drinking Chinese medicines
A2. You are willing to treat the disease by receiving acupuncture and moxibustion
A3. You are willing to treat the disease by cupping
A4. You are willing to treat the disease by doing a massage
Note: if you have not received TCM treatment, please go to A9
A5. You are satisfied with the model of diagnosis and treatment of traditional Chinese medicine
A6. You are satisfied with the therapeutic effect of Chinese medicines
A7. You are satisfied with the therapeutic effect of acupuncture and moxibustion
A8. You are satisfied with the therapeutic effect of massage
A9. You trust the therapeutic effect of traditional Chinese medicine
A10. Traditional Chinese medicine should be publicized and prompted
A11. Scientific research in traditional Chinese medicine should be strengthened

*Practice*
P1. When you suffer from chronic diseases, you will choose traditional Chinese medicine
P2. When you suffer from insomnia, you will choose traditional Chinese medicine
P3. When you suffer from diarrhea and constipation, you will choose traditional Chinese medicine
P4. When you suffer from sterility and infertility, you will choose traditional Chinese medicine
P5. When you have a bad appetite, you will choose traditional Chinese medicine
P6. When you are tired, you will choose traditional Chinese medicine
P7. When you suffer from chills and fever, you will choose traditional Chinese medicine
Note: if you are male, please go to P10
P8. When you suffer from menstruation disorder and dysmenorrhea, you will choose traditional Chinese medicine
P9. When you suffer from other gynecological diseases, you will choose traditional Chinese medicine
P10. You would like to attend the lecture about traditional Chinese medicine
P11. You would like to recommend traditional Chinese medicine to others
P12. You would like to use health tea or medicinal diet made by Chinese medicines

**Table 7:** General information of participants (*n* = 176).

Parameter	*n*	%
*Gender*		
Female	86	48.9
Male	90	51.1

*Age*		
Less than 20 years	12	6.8
20 to 39 years	103	58.5
40 to 59 years	54	30.7
More than 60 years	7	4.0

*Nation*		
America	42	23.9
England	36	20.5
Australia	35	19.9
Canada	18	10.2
India	16	9.1
Italy	8	4.5
Germany	8	4.5
Thailand	5	2.8
Pakistan	2	1.1
Korea	2	1.1
Japan	3	1.7
Iraq	1	0.6

*Education*		
Primary education and below	—	—
Secondary education	23	13.1
University education and above	153	86.9

*Profession*		
Student	47	26.7
Education	50	28.4
Medicine	19	10.8
Peasant	1	0.6
Merchant	4	2.3
Factory worker	3	1.7
Company employee	37	21.0
Housewife	12	6.8
Retiree	3	1.7

**Table 8:** Pattern matrix a of questionnaire items.

Factor					
Item		Dimension	1	2	3
When you are tired, you will choose traditional Chinese medicine					
	P6	Practice	0.946		
When you suffer from insomnia and dreaminess, you will choose traditional Chinese medicine					
	P2	Practice	0.935		
When you have a bad appetite, you will choose traditional Chinese medicine					
	P5	Practice	0.866		
When you suffer from diarrhea and constipation, you will choose traditional Chinese medicine					
	P3	Practice	0.844		
When you suffer from chills and fever, you will choose traditional Chinese medicine					
	P7	Practice	0.819		
When you suffer from sterility and infertility, you will choose traditional Chinese medicine					
	P4	Practice	0.57		
When you suffer from chronic diseases, you will choose traditional Chinese medicine					
	P1	Practice	0.554		
You would like to use health tea or medicinal diet made by Chinese medicines					
	P12	Practice	0.468		
You would like to attend the lecture about traditional Chinese medicine					
	P10	Practice	0.41		
You are willing to treat the disease by cupping					
	A3	Attitude		0.846	
Traditional Chinese medicine should be publicized and prompted					
	A10	Attitude		0.807	
You are willing to treat the disease by receiving acupuncture and moxibustion					
	A2	Attitude		0.80	
You trust the therapeutic effect of traditional Chinese medicine					
	A9	Attitude		0.791	
You would like to recommend traditional Chinese medicine to others					
	P11	Attitude		0.606	
You are willing to treat the disease by drinking Chinese medicines					
	A1	Attitude		0.587	
You are willing to treat the disease by doing a massage					
	A4	Attitude		0.569	
Scientific research in traditional Chinese medicine should be strengthened					
	A11	Attitude		0.312	
Chinese medicine is one of the treatments of traditional Chinese medicine					
	K4	Knowledge			0.831
Acupuncture and moxibustion is one of the treatments of traditional Chinese medicine					
	K5	Knowledge			0.798
Massage is one of the treatments of traditional Chinese medicine					
	K6	Knowledge			0.714
Traditional Chinese medicine is the Chinese traditional culture					
	K2	Knowledge			0.668
Cupping is one of the treatments of traditional Chinese medicine					
	K7	Knowledge			0.633
Traditional Chinese medicine is effective in treating chronic disease					
	K3	Knowledge			0.542
Traditional Chinese medicine is extensive and profound					
	K1	Knowledge			0.238

Extraction method: principal component analysis; rotation method: optimal skew method of Caesar normalization; a: the rotation converges after 5 iterations.

## Data Availability

The data came from the questionnaire survey results can be available upon request.

## References

[1] Han R. D. (2018). Chinese medical treasures of the motherland. *Hina New Time*.

[2] Lin A. X., Chan G., Hu Y. (2018). Internationalization of traditional Chinese medicine: current international market, internationalization challenges and prospective suggestions. *Chinese Medicine*.

[3] Deng L. J., Mou Y. N. (2018). Review on the internationalization of traditional Chinese medicine. *Modern Business*.

[4] Overseas Chinese group of traditional Chinese medicine (2016). Overview of international traditional Chinese medicine development and legislation. *Guiding Journal of Traditional Chinese Medicine and Pharmacology*.

[5] Lv Z. Y. (2016). Problems and countermeasures in the development of traditional Chinese medicine internationalization. *Jiangsu Traditional Chinese Medicine*.

[6] Ye S. S., Pan Z. G., Liu W. W., Zhu L. P. (2018). General practitioners’ KAP questionnaire on anticoagulation in patients with nonvalvular atrial fibrillation using delphi method. *Chinese General Practice*.

[7] McPherson S., Reese C., Wendler M. C. (2018). Methodology update. *Nursing Research*.

[8] He Y., Yang X. L. (2018). Research on evaluation index system of mental health service accessibility based on delphi method. *Chinese General Practice*.

[9] Zhao W. T., Huang Y., Zhang X. M. (2018). Investigation on knowledge, attitude and practice about prevention of venous thromboembolism in geriatric nurses. *Chinese Journal of Modern Nursing*.

[10] Shen L., Yang J., Jin X., Hou L., Shang S., Zhang Y. (2019). Based on delphi method and analytic hierarchy process to construct the evaluation index system of nursing simulation teaching quality. *Nurse Education Today*.

[11] Zhang H.-M., Bai M.-H., Wang Q. (2017). Development, reliability and validity of traditional Chinese medicine health self-evaluation scale (TCM-50). *Chinese Journal of Integrative Medicine*.

[12] Guo X., Wu X., Guo A., Zhao Y. (2018). Reliability and validity of the Chinese CECA10 questionnaire for Chinese patients with condyloma acuminata. *Medicine (Baltimore)*.

[13] Buchman S., Attia E., Dawson L., Steinglass J. E. (2019). Steps of care for adolescents with anorexia nervosa-a delphi study. *International Journal of Eating Disorders*.

[14] Wang W. J., Liu X. L., Chen B., Li C. F., Feng N. P. (2016). Development on the diabetes self-management knowledge, attitude, and behavior assessment scale; (DSKAB). *Chinese Journal of Preventive Medicine*.

[15] Chen Y. Y., Tao F. S., Jiang W. T. (2012). Application of delphi in preparation for the pre-hospital first-aid medical equipments for urban peace-keeping wounded persons. *Chinese General Practice*.

[16] Ang B. H., Chen W. S., Ngin C. K., Oxely J. A., Lee S. W. H. (2018). Reliability and validity of the English and Malay versions of the driving and riding questionnaire: a pilot study amongst older car drivers and motorcycle riders. *Public Health*.

